# A cross-sectional evaluation of binge-eating behavior and its correlation with anxiety disorders among adolescents in Northern Saudi Arabia: implications for future generations

**DOI:** 10.3389/fpsyt.2024.1384218

**Published:** 2024-11-07

**Authors:** Ahmed M. Alhuwaydi

**Affiliations:** Department of Internal Medicine, Division of Psychiatry, College of Medicine, Jouf University, Sakakah, Saudi Arabia

**Keywords:** anxiety disorders, Hamilton Anxiety Scale, binge-eating behavior, Saudi Arabia, adolescents, associated factors

## Abstract

**Background and aim:**

Binge-eating behavior and anxiety disorders pose a significant public health issue worldwide, as it has severe implications for both the physical and mental health of the adolescent population. The present study evaluated the prevalence of binge-eating behavior, anxiety disorders, and associated factors among the northern Saudi adolescent population. Furthermore, we assessed the correlation between binge eating and anxiety.

**Methods:**

The present population-based cross-sectional study was carried out among adolescents in the Aljouf region of Saudi Arabia from June 2023 to December 2023. A total of 384 eligible participants were selected using the convenience sampling method. The present study used a pretested Arabic version of the binge eating scale (BES) and Hamilton Anxiety Scale (HAM-A) to assess the binge-eating behavior and anxiety disorders among the target population. The Spearman correlation test determined the strength and direction of the correlation between BES and HAM-A scores. Furthermore, logistic regression analysis was applied to find the associated factors for binge-eating behavior among the study participants.

**Results:**

Of the 384 participants, moderate and severe binge-eating behaviors were found among 11.2% and 8.3% of the respondents, respectively. Regarding the severity of anxiety as assessed by the HAM-A scale, mild, moderate, and severe anxiety were shown among 12.8%, 9.6%, and 7.5% of the participants, respectively. Also, the study found a positive correlation between binge eating and anxiety scores, with a correlation coefficient of 0.26 and a p-value of 0.001. Furthermore, being female (p = 0.001), moderate (p = 0.004), and severe anxiety (P = 0.001) were significantly associated with binge-eating behavior.

**Conclusion:**

The present research findings advocate for the implementation of targeted interventions and support services aimed at decreasing binge-eating behavior and anxiety, thereby promoting the overall well-being of adolescents and building stronger future generations. Moreover, it is recommended that optional courses about binge eating be incorporated into the curricula of schools and universities.

## 1 Introduction

Adolescence is considered a complex and diverse period characterized by enormous physical and psychological developmental changes (1, 2). Nutritional aspects are regarded as the most susceptible phase of a lifetime, where they make eating and lifestyle choices that eventually determine the future status of their health and well-being (3). A handful of research communications have demonstrated an increased prevalence of metabolic syndrome such as obesity in adolescents, which, along with rapid social and cultural alterations, make them more vulnerable to developing numerous disordered eating behaviors like anorexia nervosa, bulimia nervosa, binge eating, frequent abstaining from eating, and partial syndromes (4, 5).

Binge eating is a disordered eating pattern characterized by consuming an excessive amount of food within a short period, often accompanied by a sense of loss of control during the binge and intense negative emotions such as guilt, shame, and distress afterward (6). This behavior poses a significant public health issue worldwide, as it has serious implications for both physical and mental health. Binge eating is a hallmark symptom of binge eating disorder, which is the most common eating disorder globally (6, 7). According to the Global Burden of Disease 2019 study, the number of individuals binge eating in 2019 alone was about 41.9 million, of which 17.3 million had binge eating disorder, and 24.6 million had other specified feeding or eating disorders. Globally, binge eating disorder and eating disorders resulted in about 3.7 million disability-adjusted life years (8). Binge eating disorder is a significant health concern that is becoming increasingly prevalent among adolescents worldwide (8, 9).

The impact of binge eating on the adolescent population is considerable, and it can also lead to several psychological disorders, including anxiety disorder, which further exacerbate the negative impact on their quality of life (10, 11). Simultaneously, anxiety disorders are one of the most common mental health issues prevalent among adolescents, according to the World Health Organization (WHO) (12). Furthermore, the coexistence of comorbid conditions, such as binge-eating behavior and anxiety disorders is substantial, demonstrating a lifetime prevalence of about 37% (13, 14). Recent epidemiological studies have confirmed that abnormal eating attitudes and behaviors are becoming more common in several Arab countries, including Saudi Arabia. As disordered eating can lead to serious consequences, it is crucial to identify these behaviors early on and provide appropriate interventions during this critical period of growth and development (15, 16). Binge-eating behavior among the Saudi population is occurring at an increasing rate and is noted as one of the significant public health problems in Saudi Arabia (17–19). For instance, a survey by Alsheweir A. et al. reported that about 30% of their study population (aged 12 to 19 years) had increased risk of having eating disorders (20). This could be due to rapid socioeconomic development in this region and lifestyle changes, such as adopting Western lifestyles and dietary habits. Studies by Melisse et al. (21) and Al Shebali et al. (22). demonstrated the roles of Westernization in eating disorders. Interestingly, Al Shebali et al. found that the eating disorders rate among their study participants was comparable to the Western standard (22), while later research by Melisse et al. did not find sufficient association (23). Similar to binge-eating disorders, anxiety disorders are also a major public health issue among adolescents due to academic pressure, family expectations, and changes in family dynamics (21, 24, 25). Some research studies have attempted to explore binge-eating behaviors in Gulf Cooperation Council countries, particularly in Saudi Arabia, and as such, the exact prevalence of binge-eating behaviors in conservative societies like Saudi Arabia could be underreported (8, 18, 21). In Saudi Arabia, around 30% are at risk for an eating disorder (26), and a study found that 18.8% of the university students at Saudi Public University have binge-eating disorders (18). A recent study in Saudi Arabia by AlHadi et al. reported that about 3% of their study population had a confirmed eating disorder (27). Interestingly, participants with binge-eating behavior demonstrated an increased likelihood of being trapped in nicotine dependency in contrast to those without binge eating disorder (18). A survey at King Abdul Aziz University in Jeddah observed a high frequency of binge eating among participants at high risk of developing an eating disorder (those with EAT-26 score ≥20) compared to the low-risk group, with 19.4% binge eating 2-3 times per month in contrast to 9.8%, respectively. Moreover, 8.2% of the high-risk cohort binge eat 2-3 times per week in comparison to 4.9% in the low-risk cohort (19). Some authors evaluated the associated epidemiological and lifestyle factors that as associated with binge-eating behaviors. Even though there are wide variations across the studies, some factors such as being female, older students, higher body mass index (BMI), smokers, and parents’ socioeconomic status were some of the factors commonly associated with the eating disorders (21, 23, 28).

Given the high prevalence of binge eating and anxiety, comprehending the intersection between the two is critical, as both illnesses can aggravate one another, producing a vicious cycle that harms an adolescent’s physical and mental health (12, 13, 29). Hence, evaluating these factors could help policymakers implement necessary intervention programs that suit cultural contexts. The increasing prevalence of mental health issues such as anxiety disorders and binge eating among Saudi adolescents emphasizes the need for urgent locally relevant and updated data. Even though some authors documented the binge-eating disorder in Saudi Arabia, there is still insufficient studies that explored the correlation between these two issues, especially in a culturally conservative society that is coupled with rapid modernization. Hence, there is an existing research gap. Moreover, ongoing assessment of the associated epidemiological characteristics is critical, as these factors are dynamic. Furthermore, there is necessity of the data related to these conditions for the guidance of clinical practice and foundations for the future research. Considering the availability of limited literature in this context, the present study aimed to evaluate the prevalence of binge-eating behavior, anxiety disorders, and associated factors among the northern Saudi adolescent population. Furthermore, the author assessed the correlation between binge eating and anxiety. Accordingly, the following hypotheses were formed for the present study.

- Binge-eating behavior and anxiety disorders are present among adolescents in Northern Saudi Arabia.
- Adolescents with anxiety disorders will have different levels of binge-eating behaviors in comparison with those without anxiety disorders.
- There will be a relationship between binge-eating behaviors and anxiety disorders.
- Differences in binge-eating behavior exist within the adolescent population based on demographic factors.

## 2 Participants and methods

### 2.1 Study description

The population-based cross-sectional study was conducted in the Aljouf region of Saudi Arabia from July 2023 to December 2023. This region is situated in northern Saudi Arabia, bordering Jordan, with a total population of about half a million. This region has four governorates: Sakaka, Qurrayat, Tabarjil, and Duma Aljindal. We included school-going and preparatory years in the adolescent population (aged 10 - 19 years) in the study. We excluded the adolescents who were physically disabled, expatriates, unaccompanied adolescents, and those parents who were unwilling to participate in the survey.

### 2.2 Sampling strategies

We determined the minimum number of required adolescent participants for this survey by employing the Raosoft online sample size calculator, which utilizes the principles outlined in Cochran’s formula (*n* = z^2^pq/e^2^) for sample size estimation (30). In this formula, *n* = minimum required participants, z = 1.96 (95% confidence interval), p = expected prevalence, q = 1-p, and e = 5% margin of error. Since the wide range of prevalence of binge eating behavior was depicted by different surveys, we have taken the expected prevalence of 50% for binge-eating behavior among adolescents, as this conservative estimate provides the maximum sample size for the study. After applying the values mentioned above in the Raosoft online sample size calculator, we determined that a minimum of 384 participants are required for the present study. The present study participants were recruited from different public places, such as shopping centers, mosques, and parks using a convenience sampling method. In order to get diverse population, the author limited the data collection to 20 adolescents per day.

### 2.3 Ethical considerations

The survey team obtained ethical clearance from the Aljouf Health Affairs Ministry of Health (wide approval no: 87-2023, dated 10 July 2023). After briefing the research purposes, we obtained informed consent from the parent (if the age is less than 18 years) or from the participant (18 years and above). Even after signing the informed consent, participants had the right to continue or refuse participation in the study. Furthermore, we adhered to the guidelines of the Declaration of Helinski throughout the study period (31). Finally, brief health education messages related to eating disorders and necessary interventions were given to all participants by the data collectors once they completed the survey.

### 2.4 Data collection procedure

The present study used a pretested Arabic data collection form to collect data from the participants. This tool is adapted by the author from the previously published and validated tools. The adapted data collection tool was translated into Arabic language using the standard translation protocol. Initially, the adapted tool was given to 35 eligible adolescents during pilot study. The average duration to complete the survey was about 10 minutes and all the respondents commented that the data collection tool was straightforward and easy to complete. The data collection form consisted of three sections.

Sociodemographic and health-related items: The first section of the questionnaire evaluated the sociodemographic and health-related details of the participants, such as age, gender, education level (present), father’s job, mother’s job, average monthly income, weight (kgs), and height (cms). We followed the standard protocols in anthropometric measurements of the adolescent population (32, 33). The BMI of the adolescents was determined using the equation: BMI = weight (kg)/height (m^2^). Adolescents with a BMI of 95^th^ percentile and above were considered obese, and from the 85^th^ to 94^th^ percentile were viewed as overweight (34). Anything other than dieting and exercising is a ‘slim technique’ used by a person to try and lose weight. Some of these techniques are taking weight loss supplements, herbal teas, and other counter products that are advertised for weight loss. Likewise, “measuring weight daily” has meant people taking their weights on an everyday basis as a way of undertaking weight loss therapy. In addition, the first part of the study questionnaire, which involves sociodemographic factors, was supposed to be filled out with the help of parents.

Binge Eating Scale (BES): The second section evaluated the participants’ eating behavior through a BES that consisted of 16 items. Every item had 3–4 separate answers, and each was given a numerical value (ranging from zero to three). The cumulative score for all responses across the 16 questions ranges from 0 to 46. A score of 27 or higher is conventionally used as a threshold for identifying severe binge eating, while a score of 17 is used to identify mild or no binge eating, and a score from 18 to 26 is considered a moderate binge eating category. The BES has reported internal consistency with Cronbach’s alpha of about 0.86 in previous studies (23, 35, 36). The pilot study also revealed Cronbach’s alpha of 0.83, which was good and acceptable.

Hamilton Anxiety Scale (HAM-A): The final section consisted of the HAM-A, which has been validated and comprises 14 elements that define the symptoms of anxiety, both psychological and somatic. Each element is rated on a scale of 0 (not at all) to 4 (very severe), with overall higher scores indicating more severe anxiety symptoms (37, 38). Earlier studies reported that the HAM-A scale can be used in various settings, including among the adolescent population (39, 40). We categorized overall HAM-A scale scores according to Matza LS et al. into no (≤ 7), mild (8 to 14), moderate (15 to 23), and severe (24 and above) (41). Like the BES, the pilot study revealed that Cronbach’s alpha value of the HAM-A scale was also good and acceptable (0.88).

### 2.5 Data analysis

The present binge-eating behavior assessment study data was analyzed with the statistical package for social science, version 24. Descriptive statistics such as counts and percentages were used for categorical variables, while mean and standard deviation were used to report continuous measures. The normality assumption of data was tested through Skewness-Kurtosis analysis. Since the test results did not meet the normality assumption, the present study applied Spearman’s correlation test to determine the strength and direction of the correlation between HAM-A and BES scores. The associated factors for binge-eating behavior were analyzed using logistic regression analysis (enter method) after adjusting for the other covariables of the study. A p-value of less than 0.05 and an adjusted odds ratio that does not include the null value were set as statistically significant values.

## 3 Results

The results of this study are organized under the following sub-headings:

### 3.1 Sociodemographic characteristics

**Table 1** reveals the sociodemographic features of the participants. The mean age of the studied adolescents was 15.23± 2.80 years. After categorizing the age group according to the best possible class interval, 33.3%, 32.6%, and 34.1% belonged to the age group of early (10 to 13 years), middle (14 to 16 years), and late adolescents (17 to 19 years), respectively. The gender proportions were almost equal, with 50.5% being males. Nearly 40% of the participants had a high school education. Regarding the parents’ occupation, 60.4% of their fathers and 51.6% of their mothers work in the governmental sector, respectively. The family’s monthly income was more than 7000 Saudi Riyals (SAR) among 53.1% of the respondents. Seven percent of the participants were smokers. Concerning BMI, 15.4% and 14.3% of the participants were overweight and obese, respectively.

**Table 1:** Sociodemographic characteristics of the adolescent population (n = 384).

Variable	Frequency	Proportion
Sex		
Male	194	50.5
Female	190	49.5
Age groups, year		
10 to 13	128	33.3
14 to 16	125	32.6
17 to 19	131	34.1
Level of Student’s Education:		
Primary school	128	33.3
Up to high school	152	39.6
Preparatory year	104	27.1
Occupation status of the father		
Government	232	60.4
Private sector	107	27.9
Unemployed	45	11.7
Occupation status of the mother		
Government	198	51.6
Private sector	97	25.2
Unemployed	89	23.2
Monthly family income		
Less than 5000 SAR	88	22.9
5000 to 7000 SAR	92	24.0
More than 7000 SAR	204	53.1
Education status of father		
University level and above	203	52.9
High school	145	37.8
No formal education	36	9.4
Education status of mother		
University level and above	237	61.7
High school	93	24.2
No formal education	54	14.1
Smoking status		
Smoker	27	7.0
Nonsmoker	357	93.0
	Mean	Standard deviation
Age	15.23	2.80
Weight of the student	55.83	15.43
Height of the of student	156.33	12.99
BMI		
Thinness	13	3.4
Normal	257	66.9
Overweight	59	15.4
Obesity	55	14.3

### 3.2 Weight management measures

**Figure 1:**
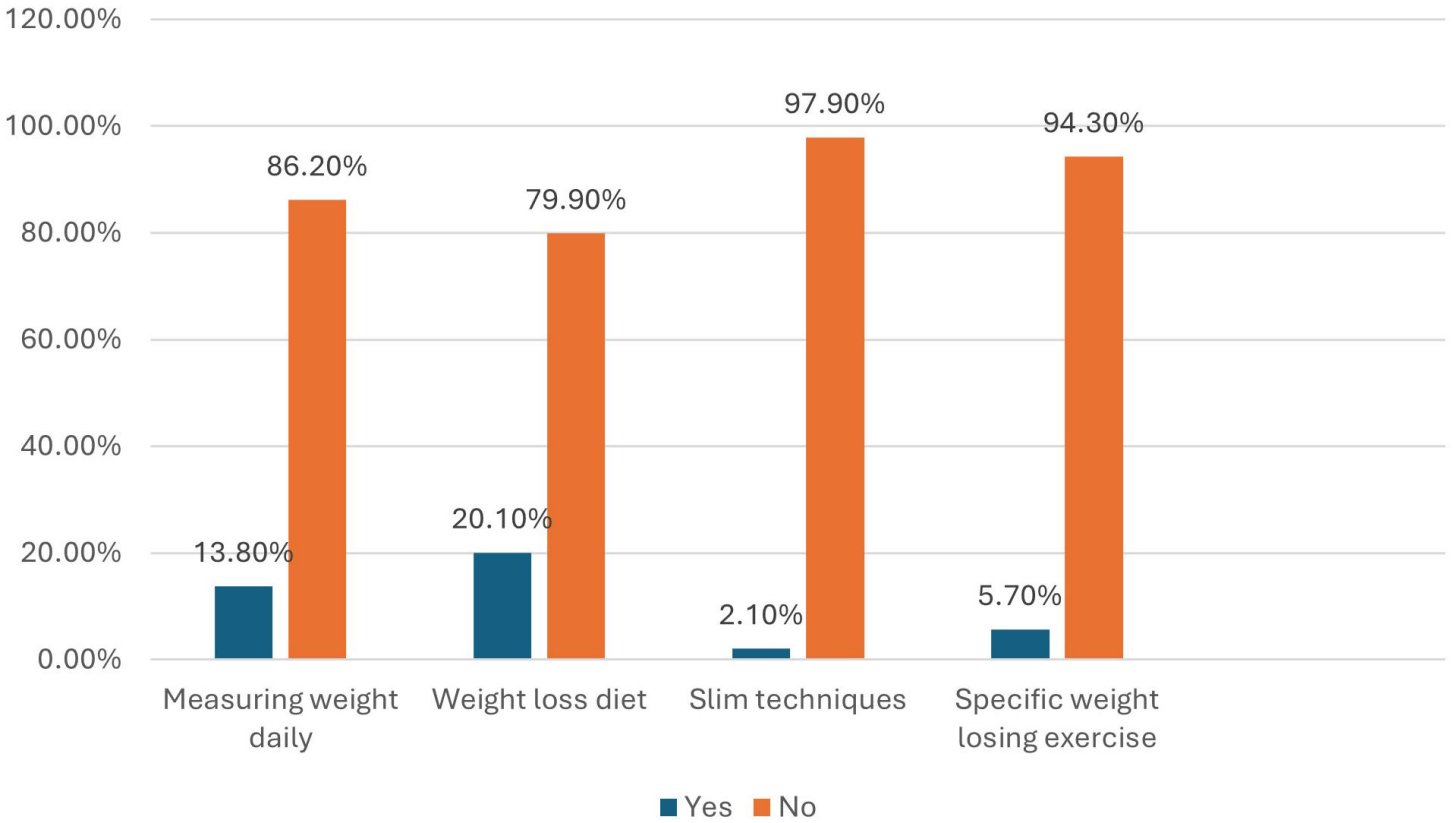
Measures taken by the participants for their weight management (n = 384).

Regarding measures taken by the participants for weight management, daily weight measurement, using diet for weight management, slim techniques, and weight-losing exercises were adopted by 13.8%, 20.1%, 2.1%, and 5.7% of the participants, respectively (**Figure 1**).

### 3.3 Binge eating behavior assessment

**Table 2** depicts the responses of the participants to the BES. Concerning eating behavior, difficulty eating slowly in the right way, having the habit of eating when feeling bored, feeling the urge to eat even when they were not hungry, and losing complete control of food when dieting was shown among 27.4%, 38%, 36.4%, and 31% of the participants, respectively. Regarding taking snacks between meals, 42.3% of the respondents reported doing this behavior. Concerning emotional cognition with binge eating, eating the same way when eating with others, thinking about food too much, and eating food upon physical hunger were reported by 38.6%, 37.6%, and 36.2% of the respondents, respectively. **Table 3** demonstrates the binge eating behavior categories. Moderate and severe binge eating behaviors were found among 11.2% and 8.3% of the respondents, respectively.

**Table 2:** Responses to the binge eating scale as reported by the studied participants (n = 384).

Variable	No problem (0)	Mild (1)	Medium (2)	High (3)
	n (%)	n (%)	n (%)	n (%)
BES (Q1): Conscious about my look and body weight	188 (49.0)	88 (22.9)	65 (16.9)	43 (11.2)
BES (Q2) Difficulty eating slowly in the right way	279 (72.7)	46 (12.0)	43 (11.2)	16 (4.2)
BES (Q3): Controlling the impulses to eat	303 (78.9)	34 (8.9)	34 (8.9)	13 (3.4)
BES (Q4): Eating when I’m bored	238 (62.0)	90 (23.4)	35 (9.1)	21 (5.5)
BES (Q5): Feeling physically hungry before eating	244 (63.5)	66 (17.2)	47 (12.2)	27 (7.0)
BES (Q6): I don’t feel any guilt or hatred for myself after I overeat*	260 (67.7)	84 (21.9)	40 (10.4)	N/A
BES (Q7): I do not lose complete control of my food when dieting, even after overeating for certain periods	265 (69.0)	61 (15.9)	47 (12.2)	11 (2.9)
BES (Q8): I rarely eat so much food that it makes me feel uncomfortably stuffed.	260 (67.7)	45 (11.7)	44 (11.5)	35 (9.1)
BES (Q9): My calorie intake level does not rise much or fall too much on a regular basis.	268 (69.8)	50 (13.0)	49 (12.8)	17 (4.4)
BES (Q10): I am usually able to stop eating when I want.	276 (71.9)	54 (14.1)	36 (9.4)	18 (4.7)
BES (Q11): I have no problem stopping eating when I feel full.	267(69.5)	69 (18.0)	33 (8.6)	15 (3.9)
BES (Q12): I seem to eat the same way when I am with others (family and social gatherings) when I am alone.	236 (61.5)	94 (24.5)	39 (10.2)	15 (3.9)
BES (Q13): I sometimes eat three meals a day with a snack.	222 (57.8)	87 (22.7)	49 (12.8)	26 (6.8)
BES (Q14): I don’t think too much about controlling unwanted incentives to eat.	262 (68.2)	52 (13.5)	40 (10.4)	30 (7.80
BES (Q15): I don’t think about food too much.	240 (62.5)	92 (24.0)	36 (9.4)	16 (4.2)
BES (Q16): I usually know if I’m physically hungry or not. I eat the necessary amount of food to satisfy myself*.	245 (63.8)	80 (20.8)	59 (15.4)	N/A

*Q6 and Q16 consisted of only three options.

**Table 3:** Binge eating behavior categories (n = 384).

Variable	Frequency	Proportion
Low (≤ 17)	309	80.5
Medium (18 to 26)	43	11.2
Severe (> 27)	32	8.3
Total	384	100

### 3.4 Anxiety assessment

Of the 384 respondents, the highest number of responses were observed in the genitourinary system (80.7%), followed by somatic (sensory) (77.9%), gastrointestinal symptoms (77.1%), and cardiovascular systems (76.8%). In contrast, the lowest “not at all” responses were observed in tension (50.3%), anxious mood (60.9), and fears (62.2%) (**Table 4**). **Table 5** shows the severity of anxiety as assessed by the HAM-A scale. Mild, moderate, and severe anxiety were shown among 12.8%, 9.6%, and 7.5% of the participants, respectively.

**Table 4:** Participants’ responses to the Hamilton Anxiety Scale (HAM-A) (n=384).

Variable	Not at all	Mild	Moderate	Severe	Very severe
	n (%)	n (%)	n (%)	n (%)	n (%)
Anxious Mood	234 (60.9)	56 (14.6)	61 (15.9)	21 (5.5)	12 (3.1)
Tension	193 (50.3)	79 (20.6)	70 (18.2)	24 (6.3)	18 (4.7)
Fears	238 (62.0)	62 (16.1)	47 (12.2)	21 (5.5)	16 (4.2)
Difficulty falling asleep	239 (62.2)	55 (14.3)	43 (11.2)	32 (8.3)	15 (3.9)
Difficulty concentrating	239 (62.2)	79 (20.6)	39 (10.2)	9 (2.3)	18 (4.7)
Depressed Mood	244 (63.5)	60 (15.6)	44 (11.5)	23 (6.0)	13 (3.4)
Somatic (Muscular)	277 (72.1)	42 (10.9)	35 (9.1)	15 (3.9)	15 (3.9)
Somatic (Sensory)	299 (77.9)	33 (8.6)	31 (8.1)	9 (2.3)	12 (3.1)
Cardiovascular Symptoms	295 (76.8)	32 (8.3)	35 (9.1)	10 (2.6)	12 (3.1)
Respiratory Symptoms	282 (73.4)	41 (10.7)	33 (8.6)	19 (4.9)	9 (2.3)
Gastrointestinal Symptoms	296 (77.1)	33 (8.6)	32 (8.3)	16 (4.2)	7 (1.8)
Genitourinary Symptoms	310 (80.7)	29 (7.6)	20 (5.2)	20 (5.2)	5 (1.3)
Autonomic Symptoms	289 (75.3)	33 (8.6)	33 (8.6)	11 (2.9)	18 (4.7)
Behavior during survey	291 (75.8)	39 (10.2)	30 (7.8)	16 (4.2)	8 (2.1)

**Table 5:** Anxiety categories according to the Hamilton Anxiety Scale (n = 384).

Variable	Frequency	Proportion
Nil (0-7)	269	70.1
Mild (8-14)	49	12.8
Moderate (15-23)	37	9.6
Severe (24 and above)	29	7.5
Total	384	100

### 3.5 HAM-A and BES correlation

The mean scores of BES and HAM-A were 21.47 ± 7.207 and 8.41 ± 9.74, respectively (**Table 6**). Spearman’s correlation showed a positive correlation between binge eating and anxiety scores, with a correlation coefficient of 0.26 and a P-value of 0.001 (**Table 7**).

**Table 6:** Total scores of binge eating scale and HAS.

Variable	All Participants (n = 384)				
	Mean	Median	Standard deviation	Skewness Statistic (Standard error)	Kurtosis Statistic (Standard error)
Total score – Binge Eating scale	21.47	19.00	7.207	1.93 (0.113)	2.77 (0.208)
Total score- Anxiety	8.41	5.00	9.74	2.51 (0.125)	3.83 (0.231)

**Table 7:** Spearman’s correlation findings between binge eating and anxiety scales.

Variable	rho*/p-value
Binge eating – Anxiety	0.26 (0.001)

### 3.6 Associated factors of binge eating behaviors

The associated factors of binge-eating behavior among the participants are presented in **Table 8**. Binge-eating behavior was significantly associated with gender and anxiety in the logistic regression analysis. Regarding sex, females were significantly at risk of binge eating compared to males (Adjusted odds ratio (AOR) = 3.312, confidence interval (CI): 1.52–5.91, P = 0.001). Moderate and severe anxiety were significantly associated with binge eating (AOR = 1.70, CI: 1.11–2.93, P = 0.004) and (AOR = 2.17, CI: 1.39–3.22, P = 0.001), respectively. Another important associated factor identified by the present study is the participants’ BMI status [obesity] (AOR = 3.31, CI: 1.93-4.87, P = 0.001).

**Table 8:** Multivariate analysis for factors associated with binge eating behaviour among the participants (n=384).

Variables	Total n= 384	Multivariable Analysis				
		Low n=309	Medium/Medium and High n=75	Adjusted odds ratio*(AOR)	95% CI	P-value
Sex						
Male	194	143	51	Ref		
Female	190	166	24	3.312	1.52 – 591	0.001
Age (in years)						
10 to 13	128	105	23	Ref		
14 to 16	125	99	26	0.708	0.34 – 1.46	0.349
17 to 19	131	105	26	1.037	0.40 – 2.71	0.940
Level of student’s Education status:						
Primary	128	105	23	Ref		
Up to high school	152	121	31	1.032	0.87 – 1.78	
Preparatory year	104	83	21	0.987	0.37 – 2.63	0.979
Monthly family income						
Less than 5000 SAR	88	77	11	Ref		
5000 to 7000 SAR	92	70	22	0.455	0.21 – 1.01	0.053
More than 7000 SAR	204	162	42	1.196	0.64 – 2.24	0.576
Education status of father						
College or more	203	162	41	Ref		
High school	145	118	27	1.103	0.36 -3.37	0.864
No formal education	36	29	7	1.304	0.44 – 3.88	0.633
Education status of mother						
College or more	237	188	49	Ref		
High school	93	75	18	1.168	0.45 – 3.02	0.748
No formal education	54	46	8	1.304	0.43 – 3.28	0.743
Occupation status of the father						
Government	232	188	44	Ref		
Private sector	107	85	22	0.789	0.33 – 1.88	0.593
Unemployed	45	36	9	0.843	0.33 – 2.17	0.723
Occupation status of the mother						
Government	198	161	37	Ref		
Private sector	97	76	21	0.819	0.40 – 1.67	0.582
Unemployed	89	72	17	1.179	0.55 – 2.59	0.682
Smoking status						
Smoker	27	23	4	Ref		
Non smoker	357	286	71	0.423	0.13 – 1.38	0.155
Anxiety category						
Nil	269	227	42	Ref		
Mild	49	38	11	1.450	0.66 – 3.12	0.353
Moderate	37	24	13	1.702	1.11 – 2.93	0.004
Severe	29	20	9	2.173	1.39 – 3.22	0.001

*Sex, age group, education status of the student, monthly family income, education status of the mother, education status of the father, occupation of the mother, occupation of the father, smoking status, anxiety category.

## 4 Discussion

Binge-eating behavior is a serious disorder in which large amounts of food are usually consumed by affected people who feel unable to stop such eating. This behavior is more common among young and middle-aged people. It leads to weight gain, type 2 diabetes mellitus, cardiovascular diseases, cancer, tooth decay, low self-esteem, and decreased quality of life (10, 42, 43). The present study aimed to identify the prevalence of binge-eating behavior and its sociodemographic correlates among Saudi children and adolescents. Furthermore, the present study determines the presence of anxiety symptoms in participants with binge eating behavior.

This study revealed that 19.5% of the respondents had medium-to-severe binge-eating behavior. Some authors explored binge-eating behavior in other regions of Saudi Arabia and other Arab countries, and they found a wide variation in the prevalence of this condition among their study population. For example, the prevalence was lower than in a study done in Palestine in which half of the participants showed symptoms of binge eating (44). Interestingly AlHadi et al. (27) from Saudi Arabia found a low prevalence and Melisse B et al. (21) found a higher prevalence of eating disorders among their study population. A study by Schulte SJ in United Arab Emirates reported a higher level (about one-third of the youths) moderate to high level binge eating problems among their study participants (45). Another study conducted by Abdulla ZARA among the young adults in the Gulf region reported about 21% of their study participants had binge eating behavior (46). The variation in the prevalence of binge-eating behavior could be attributed to different tools utilized to identify its presence. In addition, different study aims could be the cause of such variation in prevalence. The present study aimed to identify the presence of binge-eating behavior rather than make a diagnosis of binge eating disorder. The variations in the prevalence of binge eating among the studies in this region further emphasize the necessity of the present study, which is required for the practice, policymakers, and other stakeholders. The most common practices used by participants in this study for weight loss were daily weight measurement, using a diet for weight management, and weight-loss exercises. The most common methods for weight loss, as reported by Badrin et al., were restriction of food intake (42.4%), physical exercise (25.3%), slimming programs (4.7%), herbs (3.7%), and traditional medications (3.4%) (47). Lowry et al. revealed that exercise and restriction of fat intake were the most preferred methods among high school students to control their weight (48). These differences in the measures taken by the participants for weight loss highlight the need for tailored health programs that consider the unique cultural backgrounds.

Teenagers and young adults were more likely to suffer from anxiety when compared to older adults (49). In this study, 29.9% of the participants suffered from anxiety disorders. This agrees with studies carried out by Mohamad et al. (50) and Han et al. (51), with the risk of anxiety being at 29%. However, higher anxiety levels were shown by Choueiry et al., where 62.4% of the participants revealed a potential risk of having anxiety, and 28.7% of them have significant clinical anxiety (52). Furthermore, a higher prevalence of anxiety (32.8%) was depicted among Portuguese college students (53). According to HAM-A, mild, moderate, and severe anxiety levels were detected among 12.8%, 9.6%, and 7.5% of the participants in the present study, respectively. A study conducted by Abdel-Salam et al. revealed that 39.4% of the participants had severe anxiety (54). A Saudi study showed that 26.8% of Saudi youth and adolescents were affected by anxiety disorders (55). The diversity of anxiety levels among various studies could be due to differences in data collection tools, case definition, sampling techniques, or geographical locations.

Binge-eating behavior and its associated symptoms commonly affect females compared to males. This gender difference could be attributed to psychosocial factors such as social pressure for thinness among females (56). Binge-eating behavior is significantly associated with sex in the present study, with females having a higher binge eating risk compared to males, in agreement with other studies (57, 58). Hence, it is well-documented that globally, women are more at risk of having binge-eating behavior than men. The higher rate of binge-eating behavior among women could be due to a range of biological, psychological, and sociocultural factors (57, 59, 60). Binge-eating behavior is commonly associated with major mental health problems such as depression, anxiety, and stress (10, 18, 61). Badrasawi, M. M., and Zidan, S. J. revealed that higher levels of stressful life events and depression were demonstrated more among binge eaters compared to non-binge eaters (44). The current study depicted that binge-eating behavior was significantly associated with anxiety, which was consistent with other studies (44, 62, 63). Another vital predictor identified by the present study is the participants’ BMI status. Our findings are supported by previous studies (64, 65). Furthermore, it is worth mentioning that overweight and obesity are also linked to anxiety disorders. However, the causal association between these three domains is still unclear. Irrespective of the causal association, the measures taken to reduce the link between these domains can significantly alleviate the critical health effects for future generations.

The present study results offer great implications to practice, policy, and more research endeavors. The outcomes of the study reveal that prompt identification, and the treatment of binge-eating behavior and anxiety disorders in teenagers are crucial for improving the prognosis of the disorders. On the policy implication front, this has made it clear that there is the need to develop policies that will improve the mental health services for adolescents in Northern Saudi Arabia especially in the Aljouf region. Target-oriented interventions, especially among adolescents at high risk of having these conditions, must be planned from time to time. Moreover, the present research opens many opportunities for further investigation in the following ways. Certainly, further different longitudinal surveys especially concerning different social-culture aspects, should take place in different parts of Saudi Arabia.

### 4.1 Strengths and limitations

The present study measured one of the vital public mental health aspects using a standard methodology, especially in the area where limited data is available. Furthermore, the present study provided valuable data for practice, policy, and future research However, because limitations are inevitable for any study, the present study also had some. The study design, which is cross-sectional, captures data at a single point in time, hence the inability to establish causation and only observe associations. So, it was impossible to establish a temporal sequence of variables. While questionnaires are efficient, they might lack the depth and nuance that qualitative methods, such as interviews, can provide. Another drawback of this self-reported study is the possibility of recall bias, as the information was reported by the participants themselves. Moreover, the study involved participants from one geographical area in Saudi Arabia, which indicates that the results cannot be generalized.

## 5 Conclusion

The present study revealed that 19.5% of the studied participants had medium-to-severe binge-eating behavior. Mild, moderate, and severe anxiety were shown among 12.8%, 9.6%, and 7.5% of the participants, respectively. This study showed that binge-eating behavior was significantly associated with female sex and anxiety. These findings advocate for the implementation of targeted interventions and support services aimed at decreasing binge-eating behavior and anxiety, thereby promoting the overall well-being of adolescents, and building stronger future generations. Next, the introduction of more psychological and sociodemographic variables should be considered in future research. The present study portrays the importance of the institution of an educational program to increase awareness levels concerning appropriate nutritional status. Finally, it is recommended that optional courses about binge eating be incorporated into the curricula of schools and universities.

## Data Availability

The raw data supporting the conclusions of this article will be made available by the authors, without undue reservation.
